# Systems thinking in local government: intervention design and adaptation in a community-based study

**DOI:** 10.1186/s12961-023-01034-1

**Published:** 2023-09-04

**Authors:** Tiana Felmingham, Siobhan O’Halloran, Jaimie Poorter, Ebony Rhook, Cindy Needham, Joshua Hayward, Penny Fraser, Stephanie Kilpatrick, Deana Leahy, Steven Allender

**Affiliations:** 1 Global Centre for Preventive Health and Nutrition (GLOBE), Institute for Health Transformation, Deakin University, 1 Geringhap Street, Geelong, VIC 3220 Australia; 2 Barwon South West Public Health Unit, Geelong, Australia; 3 Victorian Health Promotion Foundation (VicHealth), Melbourne, Australia; 4 Monash University, Melbourne, Australia

**Keywords:** Prevention, Local government, Council, Systems thinking, Community, Adaptation, Health

## Abstract

**Background:**

Systems thinking approaches are increasingly being used by communities to address complex chronic disease. This paper reports on the VicHealth Local Government Partnership (VLGP) which sought to co-create improvements in the health and well-being of children and young people by working with local government in Victoria, Australia.

**Methods:**

The VLGP included a series of health promotion modules, aimed at creating policy, programme and practice changes across local government. One of these modules, *Connecting the Dots – creating solutions for lasting change*, aimed to build capacity for systems thinking in municipal public health and well-being planning across 13 councils. The approach was adapted and data were collected on the stimuli for, and results of, adaptation.

**Results:**

The council adapted the systems thinking approach to meet geographic characteristics, priority health issue/s and participant target group needs. Adaptions applied to workshop materials, training delivery, existing and new resources, and to align with other community-based approaches. Stimuli for adaptation included the COVID-19 pandemic, needs of children and young people, capacity of council to deliver the workshop series, and time available within the project or for the participant group.

**Conclusions:**

Systems thinking was used and adapted by councils to improve the health and well-being of children and young people and increase the voices of children and young people in decision-making. Flexible delivery is critical to ensure communities can adapt the approach to meet local needs.

## Background

Addressing the complex drivers of chronic disease is critical to successful prevention at the population level [1–3]. Evidence of the use of systems thinking that addresses this complexity in whole-of-community prevention initiatives continues to build. While systems thinking involves a diverse set of practises and methods, community-based system dynamics (CBSD) is emerging as a key approach used in prevention efforts. CBSD offers practical research and community engagement methods that support co-design in prevention, including participatory systems mapping techniques. Participatory systems mapping (or participatory modelling) has been used in a broad range of whole-of-community, stakeholder co-designed, complex prevention efforts in Australia and internationally [4–8].

A key advantage of CBSD is the flexibility to accommodate contextual differences between settings and communities [9, 10]. Trials in Australia [11, 12] and internationally [13] have exploited this flexibility as a mechanism to achieve scalability in prevention, most often with individual communities or municipalities as the unit of interest. During these trials, a prescribed set of participatory systems mapping methods were adapted and repeatedly used across large geographic areas. This approach was supported by group model building (GMB) literature [9, 10, 14, 15], which documents specific, structured activities that can be sequenced together and adapted into a complete participatory system mapping process.

While the adaptability of CBSD methods make them desirable where community/stakeholder co-design, complexity and sensitivity to local context are priorities, there is little in the literature to describe when, why and how these adaptations are being made during implementation. Adequately documenting emerging practises, the insights into what adaptations have been made in response to which stimuli and the relative successes of these adaptations, will be key to providing useful and practical guidance for practitioners.

In Victoria, Australia, the VicHealth Local Government Partnership: young people leading healthier communities (VLGP) was initiated in 2020 with the aim to build capacity within 13 local governments to take evidence-informed action to improve the health and well-being of children and young people [16]. The VLGP emphasized engagement with children and young people, and the use of systems thinking as guiding principles to understand the locally relevant drivers of children and young peoples’ health and well-being. This engagement helped to determine how to adapt implementation of the VLGP initiative to local contexts and priorities.

This paper will consider the following research questions in relation to the VLGP systems-based approaches to prevention, which aims to improve the health and well-being of children and young people in a local government setting:

- How was the approach adapted in response to community-specific contexts and needs of VLGP?
- What were perceived as stimuli for adaptation?

## Methods

### Study context

In 2020, VicHealth launched VLGP, which aimed to improve the health and well-being of children and young people by embedding their voices and perspectives into future municipal public health and well-being plans (2021–2025). The structure and objectives of VLGP are described in detail elsewhere [17].

### Community-based system dynamics in the VLGP

Eight evidenced-informed health promotion modules were designed to provide ‘how-to guides’ to deliver policy, programme and practice change. One of the modules, Connecting the Dots – creating solutions for lasting change (CtD), focused on building capacity within the council workforce to use the CBSD literature to guide identification, planning, development and delivery of prevention actions. CtD provided workforce capacity building in the early stages of the VLGP, with ongoing support systems embedded to maintain and mentor council teams throughout the life of the partnership.

The delivery of CtD involved two primary mechanisms. Firstly, council facilitation teams were introduced to, and supported to deliver, a predefined three-workshop series designed to engage and consult community stakeholders on the interconnected drivers of health and well-being for children and young people in their community. The workshops series was based on GMB literature [9] and approaches developed in previous Victorian prevention trials [11, 18]. Scripts (graphs over time, connection circles, model review and action ideas) were used to guide facilitation of the workshop process (Table 1) [14, 15, 19]. This supported council teams to utilize the GMB process to develop causal loop diagrams (CLD) that visualized the interrelationships between the complex drivers of health and well-being for children and young people from the community’s perspective. The process culminated in stakeholder identification and prioritization of potential prevention actions to support the health and well-being of children and young people.

**Table 1:** Descriptions of scripts used by council facilitation teams during delivery of the workshop series [19]

Script	Description
Graphs over time	Helps participants to frame a problem, identify variables (or drivers) and gather input that influences the topic for the workshop or modelling process. It is used at the beginning of a workshop
Connection circles	Used to identify connections between drivers, and additional drivers not identified in graphs over time. It is used after graphs over time
Model review	Gives participants an opportunity to summarize dynamic insights and stories, helps clarify ideas, capture additional information about the diagram and provide feedback. It is used towards the end of a workshop
Action ideas	Helps participants identify and prioritize actions after a CLD has been developed

**Fig. 1:**
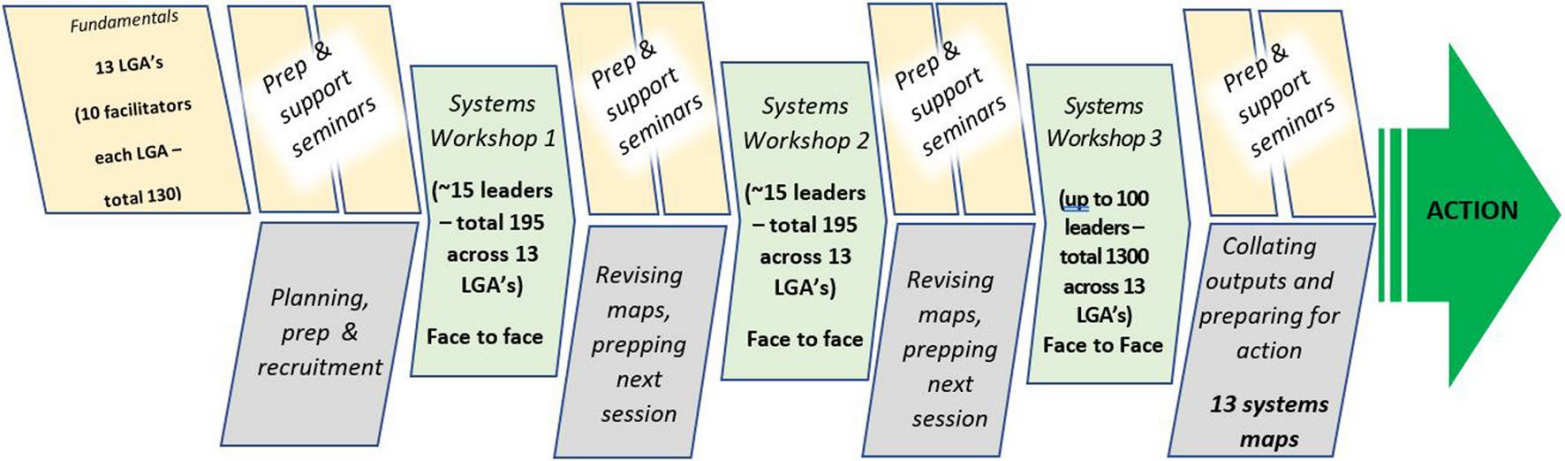
VicHealth local government partnership – connecting the dots module

Secondly, ongoing systems thinking support and mentoring from the CtD module team (academic staff experienced in the use of systems thinking in prevention, local government and community development) was provided to council teams. This included structured activities, wherein the councils used their stakeholder-informed CLD as a resource to monitor and track the sequence, progression and implementation of VLGP actions (proposed outputs are provided in Fig. 1) [20]. The broader structure of the CtD module in relation to VLGP has been described in greater detail in prior publications [17].

To develop a key problem statement to guide localized action, councils identified priority areas based on:

- age cohorts; under 25 years, those aged between 12 and 25 years, those under 12 years, or another specific age cohort
- geographic location
- target areas of health and well-being for children and young people in their community; healthy eating, physical activity, mental health or a combination.

Council staff invited workshop participants from their communities and included young people, children, parents, community health services, community-based nongovernment organizations, sports clubs, state-wide organizations or schools.

In some instances, the format or content of the workshops were adapted to the local context, which included combining content across workshops, for example, combining workshop 1 with workshop 2, to be delivered together as one session. Where this has occurred, results are reported under ‘Combined workshop 1 and 2’, or ‘Combined workshop 2 and 3’.

### VLGP leading the way – engaging young voices for change module

CtD was offered alongside an additional module, *Leading the Way – engaging young voices for change* (LtW) module which included two implementation actions. The second of these was ‘Implementation Action 2: Including children and young people in planning’. This action was delivered in the form of an online toolkit, Kids Co-designing Healthy Places (KCDHP), which included steps for children to complete a community audit and participate in a co-design workshop [21]. In some instances, where councils were working with similar cohorts across modules, councils had the option to modify content to reflect the aligning outcomes of both CtD and LtW modules. The LtW module will be described in further detail in forthcoming publications.

### Data sources and analysis

Data was collected from each of the 13 councils over a 12 month period during 2021 and 2022. Data sources included a register to track adaptations to modules and workshop content which included merging activities across modules and the reasons why. Data for the register was collected in an excel spreadsheet as part of observational project progress notes. This was informed by the unfolding, practical experiences of regional advisors as part of the project and through conversations and planning meetings with council teams. Observational statements were then coded and themed to understand experiences across the project cohort. Regional advisors and council teams regularly collected data as part of their roles, with the potential for the information to be used for other purposes beyond the scope of this module. Other data included the number of workshops, number and type of participants, type of workshop, adaptations implemented and stimuli.

Inductive thematic analysis [22] was conducted by JP, ER and TF to identify codes and themes from the adaptations and the stimuli. Coding and theming was performed independently, and any discrepancies resolved through group discussion and an agreed consensus.

## Results

Thirteen councils from across Victoria participated in the CtD module. One hundred and eleven staff from councils and partner organizations participated in the initial CtD workshop series to build capacity in the delivery of community workshops.

Of the 13 participating councils, 3 were in metropolitan areas and 10 were in rural or regional areas. Five councils focused on the well-being of children and young people aged under 25 years, five focused on those aged between 12 and 25 years and three focused on those under 12 years. Eleven councils used approaches that included their whole local government geographical area. Eight councils combined all three priority areas (healthy eating, physical activity and mental health) with a general focus on the well-being of young people and children, one council focused on healthy eating and physical activity combined, and four councils addressed mental health (with a focus on social connection).

All councils delivered at least three workshops, either standalone or in combination, that is, where content for two workshops was combined and delivered as part of one session. Workshop participants and delivery is presented in Table 2. Across most workshops, the number of young people who participated was greater than the number of organizational stakeholders, except when workshops 2 and 3 were combined. Workshops 1–3 were delivered both online and face to face, although the delivery of workshop 3 was more often face to face. Only a small number of workshops were delivered using the predefined workshop format and content, where scripts, resources and delivery were not adapted.

**Table 2:** Summary of total number of workshop participants and workshop format from Connecting the Dots [17]

	Workshop 1	Workshop 2	Workshop 3	Combined workshop 1 and 2	Combined workshop 2 and 3
Participants (*n*)					
Young people	174	99	128	52	9
Stakeholders	113	72	90	7	20
Combination of young people and organizational stakeholders			83		
Total participants	287	171	301	59	29
Workshop (*n*) format and delivery					
Implemented using the predefined format and content	2	1	3	NA	NA
Adapted	14	12	10	3	2
Delivered face to face	9	6	11	2	2
Delivered online	7	7	2	1	
Total number of workshops	16	13	13	3	2

When the total number of participants across all councils were combined, workshop 3 had the highest total number of participants (*n* = 301). There were 287 participants at workshop 1 in total, and 171 participants at workshop 2.

The total number of workshops delivered (*n* = 47) was higher than originally anticipated (*n* = 39), where several councils delivered more than the recommended three workshops (as described previously, based on GMB literature with graphs over time, connection circles, model review and action ideas scripts). In some cases, councils delivered four or five workshops across their selected geographical area, for example, multiples of workshop 1, 2 or 3.

Following the completion of workshop 3, 13 CLDs were created; one from each of the participating councils (see O’Halloran et al. for an example [17]), and each was derived specifically by the participant cohort (for example, young people, children, parents or organizational stakeholders), area of well-being (for example, healthy eating, physical activity, mental wellbeing or all three combined) and local demographics (for example, rural or metropolitan).

Five themes emerged which described the adaptations implemented across all workshops, and five themes emerged to describe stimuli for adaptation (Table 3). Where councils adapted multiple components or identified multiple stimuli, more than one code was applied. For example, if a change to a script included the development of a new resource, both ‘change to script’ and ‘changes to resources’ codes were applied.

**Table 3:** Adaptations and stimuli that influenced workshop content, by workshop and theme

	Workshop 1	Workshop 2	Workshop 3	Workshop 1 and 2	Workshop 2 and 3
Adaptations made to workshop content or delivery					
Changes to the recommended script	6	7	4	2	2
Changes to presentations	4				
Changes to delivery	7	10	9	2	2
Changes to resources	2	3	1		1
Content adapted to complement delivery alongside another module (KCDHP)	1	1	2		
Stimuli for adaptation of workshop content					
COVID-19 pandemic	7	7	6	1	2
Participant cohort	8	7	8	2	2
LGA capacity	2	1	3		
Time	1	2	5	1	
Geography	1	2	1		

Changes to delivery was the most common adaptation (Table 3). Workshops 2 and 3 had the greatest number of changes made to delivery, ten and nine respectively. A number of councils noted changes made to recommended scripts. Changes were made to the recommended steps for scripts during workshop 1 (*n* = 6), workshop 2 (*n* = 7) and workshop 3 (*n* = 4), for example, graphs over time, connection circles, model review or action ideas. Where workshops had been combined, scripts were almost always adapted (four out the five combined workshops). There were four instances where the CtD workshop content was adapted to complement the delivery of the KCDHP toolkit. This occurred once each in workshops 1 and 2, and twice in workshop 3.

The most common stimulus for adaptation across all three workshops were the COVID-19 pandemic and changes made to meet the needs of the participant cohort. Most combined workshops (*n* = 4) made changes to meet the needs of the participant cohort. Workshop 3 had the highest number of stimuli noted across all workshops. Workshops 1 and 2 had a lower number of changes made as a result of participant cohort, council capacity and time than workshop 3. Adaptation and stimuli themes that emerged through the inductive thematic analysis process, and descriptions are summarized in Table 4.

**Table 4:** Adaptation and stimuli themes that emerged through the inductive thematic analysis process, and descriptions

Themes	Description
Adaptation theme	
Changes to presentations	Changes to slides/presentation for reasons other than a pure change in script, for example, where a new slide was added, or an existing slide was deleted from the recommended slide deck
Changes to the recommended scripts	Includes new, altered or removed activities or resources, for example, where factors were identified verbally from the participant group rather than using the graphs over time script, or prioritization of action was removed from the action ideas script
Changes to delivery	Changes to the workshop format, the total number of workshops in the series, engagement methods, changes in the workshop process
Changes to resources	Where new or modified resources were developed to complement workshop content
Content adapted to complement delivery alongside another module (Leading the Way – engaging young voices for change)	Changes to workshop content to combine with content from the KCDHP toolkit
Stimuli theme	
COVID-19 pandemic	Including lockdowns, state or local restrictions (for example, number of people in a room), individual anxiety about attending group events or occurrence of infection
Participant cohort	Meeting the needs of the participant group, for example, where changes were made to ensure age appropriateness or availability
Council capacity	Including changes in staff, level of leadership support, placement of the project within the council, and confidence and ability of project staff
Time	The amount of time participants had available or project timelines from funders
Geography	Geographical characteristics of the local government area, including size, access to services and population demographics in the geographic region

At the completion of workshop 3, all 13 councils had identified a group of community-led ideas that aimed to improve the health and well-being of children and young people in their communities. The final systems map for each council, and the implementation of prioritized actions will be reported in a future study.

## Discussion

### Main findings

This study shows the need for, and the ability to, adapt and refine GMB and CBSD methods to meet the needs and changing contexts of differing local communities. Across the 13 Victorian councils participating in the CtD module, the target age of children and young people, the geographic area and the priority health issue varied. This may have been influenced by concerns about over-consultation in smaller communities, particularly where other consultations had taken place.

Throughout 2021, Victoria suffered a series of social and economic shocks related to the continuing COVID-19 pandemic [23, 24] and the lingering impacts of the Black Summer bushfires [25]. This created a highly dynamic and reactive environment within which the VLGP communities were subject to several, often sudden, changes in organizational and community priorities, opportunities for community engagement, changes in the local prevention workforce (including staff turnover) and available resources, among other factors. This was reflected in our findings, where all 13 councils delivered the content in the three-workshop series, but a number of adaptations were made to overcome challenges associated with COVID-19 to meet the needs of the participant cohort, and to allow for council staff capacity, time and geographic location. The most common adaptations were made in the delivery format across all workshops, whilst others made adaptations to the recommended script, presentations and resources, or aligned delivery structure with the LtW module and KCDHP toolkit.

Existing systems thinking approaches provide examples of how communities can be part of leading change across different complex health problems [8, 26–35]. Each have used community-based systems approaches, including adaptation where needed, to meet the needs of workshops participants and the wider community, with evaluation of the method also consistently building, as documented in a recent systematic review [36]. Adaptation of scripts is an important and expected element of GMB workshop delivery [9, 15]. The adaptation of GMB methods to meet the needs of the local community has been highlighted in earlier research [37, 38]. In an Australian study assessing the value and acceptability of the GMB methodological approach with Aboriginal communities, Aboriginal staff that participated in three GMB workshops were interviewed [37]. Findings indicated that GMB was a useful tool; however, participants suggested adapting the language, visual tools (that is, artwork) and workshop activities to enhance the relevance and cultural safety of the method [37]. Stronger engagement with Aboriginal people in the development, delivery and leadership of research projects using GMB methods was another consideration given for future work [37]. Integration of culture into the GMB process has also been demonstrated in New Zealand in the delivery of GMBs where workshops followed Māori tikanga (protocol), incorporated a karakia (Maori prayer or blessing) and a shared meal and activity designed to assist with whakawhanaungatanga (relationship building within the group) [27]. Our research supports the findings in these studies, such as encouraging a flexible environment that allows for various adaptations to be made to meet the needs of each community.

Stimuli were coded independently of each adaptation and thus our study cannot infer relationships between adaptations identified and stimuli themes, as specific relationships between the two were not captured during data collection. However, anecdotally, there were reports that redeployment of council staff and restructuring of the prevention workforce into recovery and response roles related to either or both bushfires and COVID-19 may have influenced adaptations. Findings from other studies also indicate there may be links between online delivery and the impact of the COVID-19 pandemic, including related restrictions. The impact of COVID-19 has also been sighted as a stimulant for re-orientating the GMB workshop to be delivered online in other studies, where advantages included increased number and diversity of workshop participants regardless of location, reducing travel time, cost and carbon emissions [38, 39].

### Limitations

The varying nature of workshop delivery proved to be a challenge in terms of data collection for our study. This limitation, and the varied context in which councils operate, made it difficult to draw conclusions about adaptations made across different councils. Systems thinking concepts can also be a challenge to those new to the field, coupled with the breadth of VLGP, this may have proved difficult for new staff. While our study captures adaptations, it does not capture data about those approaches that did not make adaptations. For example, of the six workshops that delivered content as recommended, no data were collected on why this occurred, nor considered any benefits or challenges to delivering content as intended.

### Strengths

Strengths include the iterative nature of workshop delivery from the outset, which allowed and encouraged council staff to adapt workshop content and delivery modes to best meet the needs of their communities. This flexibility enabled staff to create new, often novel, components that complemented GMB scripts and the recommended presentations, for example, the development of new resources for participants groups. The use of three reviewers to identify themes for adaptation and stimuli provided rigour and reduced bias during the theming process.

### Future research

Future research should ensure flexible delivery options are built into GMB project design, including the opportunity for community members to be involved in shaping key decisions influencing design, delivery and adaptations along the way. Adaptation of delivery and scripts is recommended by Hovmand [9], with its importance reiterated in our study. Adaptation of GMB content and delivery is critical to the success of systems practice in community settings. Additional documented examples of adaptations made to GMB content and delivery would also help inform future implementation of the method. Our study documented examples of communities creating their own resources to supplement GMB scripts. Future research could explore a deeper examination of resources created, including descriptions of their application, context and evaluation to provide insights into effectiveness.

## Conclusions

The CtD module is one example of building the capacity of communities to deliver participatory GMB workshops within their own communities and observing the need for the process to be flexible and adaptable. Given the vastly different contexts, including geographical and organizational contexts, and temporal demands (that is, COVID-19) each LGA was facing, their ability to deliver GMBs should be commended. Capturing the adaptations that occurred across these 13 LGAs will be critical to the evaluation of the CtD module and inform future delivery.

## Data Availability

The datasets used and/or analysed during the current study are available from the corresponding author on reasonable request.

## Abbreviations

VLGP: VicHealth Local Government Partnership
CBSD: Community-based system dynamics
GMB: Group model building
CtD: Connecting the Dots – creating solutions for lasting change
CLD: Causal loop diagrams
LtW: Leading the Way – engaging young voices for change
KCDHP: Kids Co-designing Healthy Places
